# Lysozyme as the anti-proliferative agent to block the interaction between S100A6 and the RAGE V domain

**DOI:** 10.1371/journal.pone.0216427

**Published:** 2019-05-09

**Authors:** Md. Imran Khan, Deepu Dowarha, Revansiddha Katte, Ruey-Hwang Chou, Anna Filipek, Chin Yu

**Affiliations:** 1 National Tsing Hua University, Chemistry Department, Hsinchu, Taiwan; 2 Graduate Institute of Biomedical Sciences and Center for Molecular Medicine, China Medical University, Taichung, Taiwan; 3 Department of Biotechnology, Asia University, Taichung, Taiwan; 4 Laboratory of Calcium Binding Proteins, Nencki Institute of Experimental Biology Polish Academy of Sciences, Warsaw, Poland; Russian Academy of Medical Sciences, RUSSIAN FEDERATION

## Abstract

In this report, using NMR and molecular modeling, we have studied the structure of lysozyme-S100A6 complex and the influence of tranilast [N-(3, 4-dimethoxycinnamoyl) anthranilic acid], an antiallergic drug which binds to lysozyme, on lysozyme-S100A6 and S100A6-RAGE complex formation and, finally, on cell proliferation. We have found that tranilast may block the S100A6-lysozyme interaction and enhance binding of S100A6 to RAGE. Using WST1 assay, we have found that lysozyme, most probably by blocking the interaction between S100A6 and RAGE, inhibits cell proliferation while tranilast may reverse this effect by binding to lysozyme. In conclusion, studies presented in this work, describing the protein-protein/-drug interactions, are of great importance for designing new therapies to treat diseases associated with cell proliferation such as cancers.

## 1. Introduction

Lysozyme is a universal antimicrobial polypeptide [1,2] (EC 3.2.1.17 found by Alexander Flemming). It exhibits N-acetylmuramoyl-hydrolase or muraminidase activity that breaks down the bacterial cell wall by catalyzing hydrolysis of the (1–4) β glycosidic bond [3,4]. On the basis of amino acid sequence and biochemical features three types of lysozyme have been reported: goose (g-type), conventional or hen (c-type), and invertebrate (i-type). The enzyme is also present in phagocyte-like cells of non-mammalian organisms which suggests that it acts in host security through the animal kingdom [5–7]. Chicken and human lysozyme is composed of 129 amino acids. Within the molecule there are four disulfide bonds and six tryptophan residues. Interestingly, the structure of both orthologs is very similar. Human lysozyme is produced by a diversity of exocrine glands and secreted into the relevant body fluids [8,9] but also by tissues and myelomonocytic lineage [8–10]. Lysozyme has been known as a component of the antibacterial defense pathway related to the monocyte macrophage system [11]. It has been shown that lysozyme acts as an anti-proliferative protein against human gastric cancer cells and lung fibroblast [12]. Anti-proliferative effect of this enzyme has also been reported for tumor LNCap and A549 cells [13], endothelial, ECV304, cells [13,14], breast cancer cells [15] and peripheral blood lymphocytes [15]. Most recently, it has been found that lysozyme exhibits anti-HIV1 activity [16–20]. It is also known that the enzyme exhibits strong anti-proliferative properties in the form of self-assembled nanostructure particles.

S100A6 (originally known as calcyclin) is a Ca^2+^-binding protein belonging to the S100 family. S100A6 is obviously a cytoplasmic protein, but has also been detected in the extracellular matrix and physiological fluids [21]. Gene encoding S100A6 has been identified on the basis of cell cycle–dependent appearance of its product [22]. During the transition from G_0_ to S phase of the cell cycle, the highest expression of S100A6 gene was observed. Regarding the S100A6 protein, it has been originally purified from Ehrlich ascites tumor cells [23,24]. Later, it appeared that the S100A6 protein is present in various cells and tissues and that its particularly high expression takes place in fibroblasts and epithelial cells. Moreover, S100A6 ortholog has also been isolated and partially sequenced from smooth muscle of chicken gizzard. S100A6 interacts with many ligands, among them with lysozyme and receptor for advanced glycation end products (RAGE) [25,26], which is important in view of the present work.

Cell death and proliferation are the major physiological processes that regulate tissue homeostasis. To control cell proliferation and inhibit cancer growth different agents including herbs are used. It should be also noted that controlling cell proliferation is important for treatment of some diseases. For instance, Alzheimer (AD), Huntington (HD) and Parkinson (PD) diseases can be treated by inducing cells to proliferate while some others such as premalignant diseases and cancers—by inhibition of cell proliferation [27–31].

Since it has been known that lysozyme interacts with S100A6 [25], in this work, using NMR and molecular modelling, we investigated the structure of the lysozyme-S100A6 complex and the influence of tranilast [N-(3, 4-dimethoxycinnamoyl) anthranilic acid], an anti-allergic drug, on the formation of this complex, and finally on the interaction between S100A6 and the V domain of RAGE. Since the S100A6-RAGE interaction has influence on cell proliferation [26] we then analyzed, using WST1 assay, the role the examined proteins and of tranilast on proliferation of SW480 tumor cells [32–35]. Thus, our studies might be very important in designing new therapies to treat diseases associated with cell proliferation.

## 2. Materials and methods

### 2.1 Materials

Tranilast was obtained from Sigma. Plasmid encoding mS100A6 was obtained from Mission Biotech Company. CM (carboxymethyl) Sepharose Fast Flow was purchased from GE Healthcare Life Sciences. ^15^NH_4_Cl, and D_2_O were purchased from Cambridge Isotope Laboratories. Triton X-100, β-mercaptoethanol and DTT were purchased from Water Stone Technology. The Centricon and Amicon membranes were purchased from Millipore. All other chemicals used in this study were of the highest analytical grade quality. All buffers were prepared using milli-Q water and were filtered using a 0.22-μm antiseptic filter. The SW480 cell line (CCL-288) was purchased from American Type Culture Collection (ATCC).

### 2.2 Purification of lysozyme

Plasmid encoding lysozyme was purchased in the form of bacterial stab from Addgene. Recovery of bacteria was done by spreading on an agar plate with an appropriate antibiotic (ampicillin). The plasmid was isolated using the plasmid extraction kit (Presto Mini Plasmid Kit). The cDNA encoding lysozyme was sub-cloned into the pET11a expression vector. Labelled protein was expressed in *Escherichia coli* strain BL21(DE3) grown in M9 minimal medium containing ^15^NH_4_Cl while unlabeled protein—in bacteria grown in Luria broth (LB) medium. Bacterial cultures were incubated overnight at 37°C until OD_600_ reached 0.8–0.9. Protein synthesis was induced using IPTG at 2 mM final concentration followed by 3-hour incubation. After that cells were broken by sonication in buffer containing 25% sucrose, 1 mM EDTA, and 50 mM Tris, pH 7.5, and centrifuged at 9000 rpm (Thermo Scientific, Heraeus Multifuge X3R). The supernatant was discarded while the pellet was washed with the same buffer and then with buffer containing 1% Triton X-100, 1 mM EDTA, and 20 mM Tris, pH 7.5. The resulting inclusion bodies were dissolved in buffer containing 0.1 M DTT, 50 mM NaCl, 5 mM EDTA, 8 M urea, 20 mM Tris, pH 7.5 and injected onto a CM Sepharose Fast Flow (weak cation exchanger) column, pre-equilibrated with a buffer containing 5 mM 2-mercaptoethanol, 50 mM NaCl, 1 mM EDTA, 4 M urea, and 50 mM Tris, pH 7.5. Lysozyme was eluted with a linear gradient of NaCl (0–300 mM) in the above buffer. Collected fractions containing lysozyme were concentrated up to 6–8 ml with the help of centrifugal filter devices (Amicon Ultra 15, 10000 NMWL). The lysozyme was dialyzed against deionized water at pH 2.0, and purified by a linear gradient of acetonitrile-to-water with 1% trifluoroacetic acid using reversed-phase HPLC. Finally, lysozyme was dialyzed against the NMR buffer containing 5 mM CaCl_2_, and 10% D_2_O at pH 7.0.

### 2.3 Purification of mS100A6

In NMR studies to keep the protein in a reduced state, Dithiothreitol (DTT) is used. Since lysozyme forms 4 disulfide bonds, which are cleaved in the presence of DTT, this reducing agent was omitted. Thus, to study formation of the S100A6-lysozyme complex we purified mutated S100A6 in which a cysteine residue was replaced by serine (C3S). The sequence encoding mS100A6 (C3S), shortly named mS100A6, was inserted in the pET-20b (+) T7 expression vector and expressed in *Escherichia coli* BL21(DE3) strain. Subsequent purification steps were performed as reported earlier [26,36].

### 2.4 ^1^H -^15^N HSQC NMR titration

A Varian 700 MHz NMR cryogenic spectrometer was used for the HSQC titrations at 25°C. All samples were dissolved in the NMR buffer containing 5 mM CaCl_2_, 10% D_2_O, pH 7.0. The HSQC titrations were performed by adding unlabeled mS100A6 to ^15^N labeled lysozyme at a 1:0, 1:0.33, 1:0.66 and 1:1 molar ratio. A second titration was performed by adding ^15^N labeled mS100A6 to unlabeled lysozyme at a 1:0 and 1:1 molar ratio. Lastly, tranilast was added to ^15^N labeled lysozyme at a molar ratio 1:0, 1:0.33, 1:0.66 and 1:1. The HSQC spectra were purposely overlapped to monitor differences in intensities and shifts of the cross peaks. The NMR data were processed using VNMRJ 2.3 program and further analyzed by sparky 3 [37].

### 2.5 Molecular docking of lysozyme with mS100A6 and tranilast

To create the model of lysozyme-mS100A6 and lysozyme-tranilast complexes, we used the HADDOCK (High Ambiguity Driven biomolecular DOCKing) program (version 2.2) [38–41]. The structures of lysozyme and mS100A6 were obtained from PDB ID: 6LYT and 1K9K, respectively, and the 3D structure of tranilast was taken from the Drug Bank database (ID: DB07615). The docking study and successive refinement were carried out using a number of ambiguous-interaction restraints (AIRs) and the HSQC spectra were analyzed to reveal cross peaks with significantly decreased intensities [42]. The structure of lysozyme-mS100A6 and lysozyme-tranilast complexes was selected from the first cluster with the lowest energy, and illustrated and displayed using PyMOL software [43].

### 2.6 Measurement of dissociation constant—fluorescence method

To calculate the K_d_ value we used a Hitachi F-2500 fluorescence spectrophotometer and proteins/drug in a buffer containing 5 mM CaCl_2_, 10% D_2_O, pH 7.0 [44]. Since mS100A6 does not have any tryptophan and lysozyme contains six such residues we excited the fluorescence of lysozyme at 305 nm [45]. mS100A6, at increasing concentration (from 0–11.9 μM), was added to lysozyme (3.6 μM concentration) and emission spectra were recorded at 340 nm. Data were plotted as the total concentration of the mS100A6 against the relative intensity, and nonlinear curve was plotted using the software GraphPad Prism 8 to calculate K_d_ from the equation (1) given below [46].

$$f=\frac{({\left[P\right]}_{T}+{\left[L\right]}_{T}+[K_{d}])-\sqrt{{\left({\left[P\right]}_{T}+{\left[L\right]}_{T}+[K_{d}]\right)}^{2}-4{\left[P\right]}_{T}{\left[L\right]}_{T}}}{2{\left[P\right]}_{T}}$$ Eq (1)

In Eq (1), f represents the fractional change, K_d_ for dissociation constant, whereas [P]_T_ and [L]_T_ representing the total concentration of lysozyme and mS100A6. The same method was used to measure K_d,_ of the complex with tranilast.

### 2.7 WST1 assay

The WST1 assay, performed according to the manufacturer protocol, was used to monitor the effect of examined proteins and tranilast on cell proliferation. Colorectal cancer derived SW480 cells, which produce a high amount of RAGE comparing to other cell types (Lovo,Colo205, SW620, and HCT116 cell lines) [47] were seeded at a density of 5×10^3^ cells/well, in a 96-well plate. 24 hours later, medium with 0.1% bovine serum albumin was added and cells were cultured for another 24 hours. The serum-starved cells were then incubated with 10, 50, and 100 nM mS100A6 with or without lysozyme (100 nM) and cultured for additional 48 hours. Then, to each well containing 100 μL of culture medium, 10 μL of the WST1 reagent was added and cells were cultured for 4 hours at 37°C, and then for 10 minutes at 37°C with slight agitation. Absorbance was recorded using synergy 2 micro-plate reader at 450 nm [42]. The proliferation of SW480 cells was analyzed in the same way in the presence of tranilast added at 100 nM concentration. All experiments were repeated 3 times and the results of quantitative analysis are presented as means ± SEM. Differences in mean values were tested by Student’s *t*-test. The level of statistical significance was set at *p* ≤ 0.05.

## 3. Results and Discussion

### 3.1 Determination of interacting site of labeled lysozyme with mS100A6

**Fig 1:**
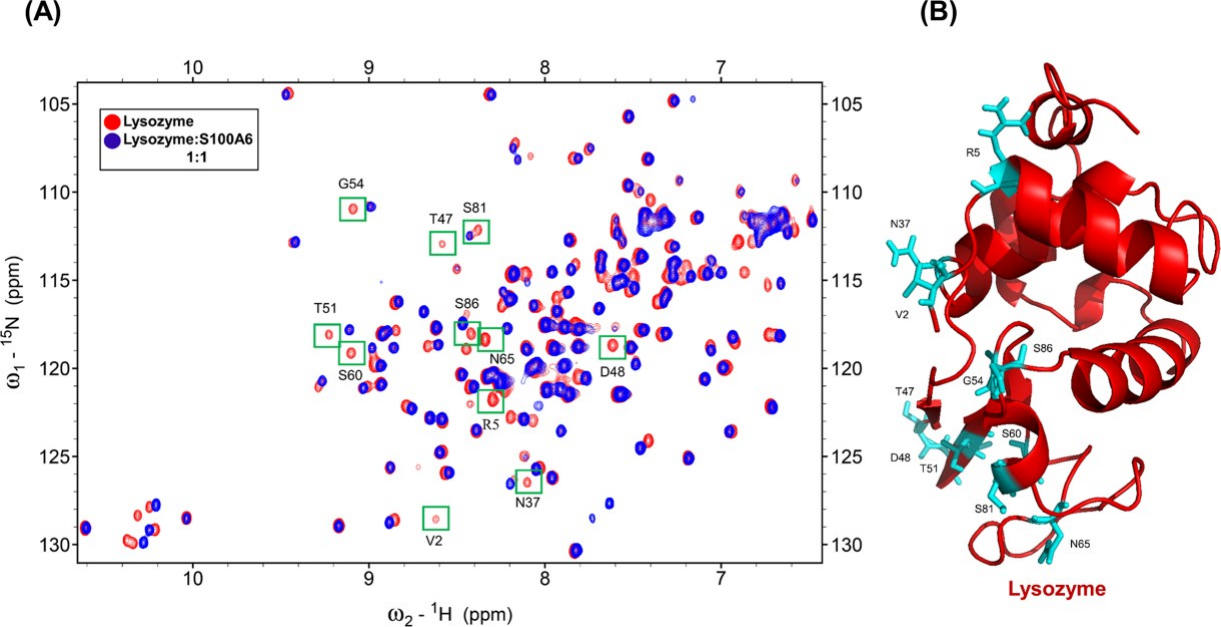
(A) Overlapped HSQC spectra of ^15^N-labeled lysozyme (red) and of ^15^N-labeled lysozyme with mS100A6 (blue). Disappearing cross peak intensities which represent interacting amino acids (V2, R5, N37, T47, D48, T51, G54, S60, N65, S81 and S86) are boxed in green. (B) The 3D ribbon diagram of lysozyme (red) shows residues interacting with mS100A6) in sticks form (cyan).

To find the binding site of S100A6 on lysozyme we used 2D NMR HSQC titration and the reported assignment of lysozyme at pH 3.8 [48,49]. Fig 1A shows the HSQC spectra of ^15^N labeled lysozyme (red) overlapped with the HSQC spectra of ^15^N labeled lysozyme complexed with unlabeled mS100A6 (blue). As it can be seen in this Fig 1A there are differences in NMR HSQC spectra (including complete disappearing of some cross peak intensities) after addition of mS100A6 to the lysozyme. This is, most probably, due to the interaction between lysozyme and mS100A6. Few residues are not taken as interacting residues because they appeared as we increased the Ph from 3.8 to 7, and some of them are side chain.

### 3.2 Determination of interacting site of labeled mS100A6 with lysozyme

**Fig 2:**
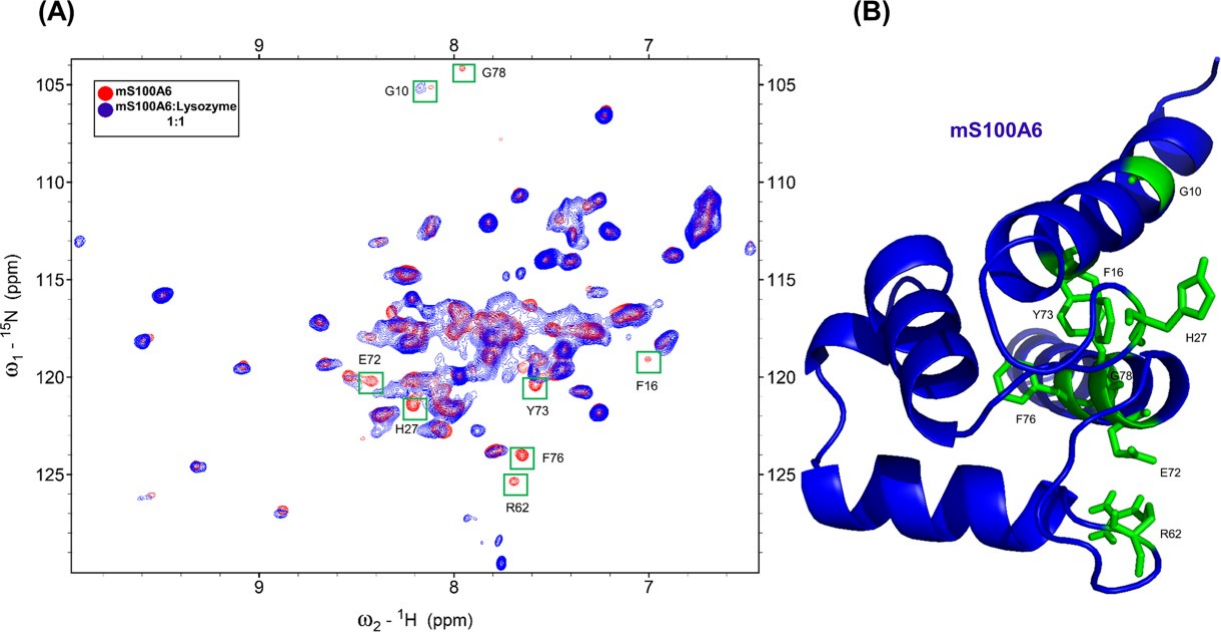
(A) Overlapped HSQC spectra of ^15^N-labeled mS100A6 (red) and of ^15^N-labeled mS100A6 with unlabeled lysozyme (blue). Disappearing cross peak intensities which represent interacting amino acids (G10, F16, H27, R62, E72, Y73, F76 and G78) are boxed in green. (B) The 3D ribbon diagram of the monomer mS100A6 (blue) shows residues interacting with lysozyme in sticks form (green).

The same method was also used for the HSQC titration of mS100A6, applying the assignment reported earlier [26,50]. In Fig 2A, the HSQC spectra of ^15^N labeled mS100A6 (red) overlapped with spectra of the ^15^N labeled mS100A6 complexed with unlabeled lysozyme (blue). The differences in NMR signals (including disappearance of some cross peak intensities) after addition of lysozyme to mS100A6 are observed. The HSQC signals of labeled mS100A6 bound to lysozyme at the interacting site are significantly lower than those of free mS100A6. This is, most probably, due to direct interaction between lysozyme and mS100A6.

### 3.3 Docking study of lysozyme with mS100A6

To generate the protein-protein interaction in the lysozyme-mS100A6 complex the HADDOCK 2.2 software was used [38–41]. Ambiguous interaction restraints (AIR) were acquired from the residues for which cross peak intensities disappeared in the HSQC spectra of the lysozyme-mS100A6 complex. Those residues provided the amino acid restraints as the input parameters for the calculated structure of the complex in HADDOCK. The structures of lysozyme and Ca^2+^-bound mS100A6 were taken from PDB IDs: 6LYT and 1K96, respectively.

**Fig 3:**
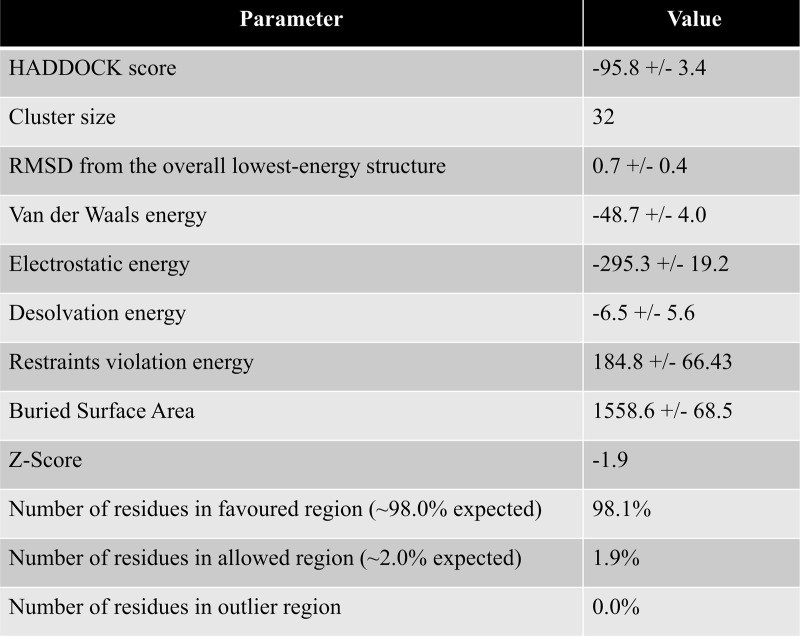
Haddock calculated parameter of the lysozyme-mS100A6 complex of cluster 1.

**Fig 4:**
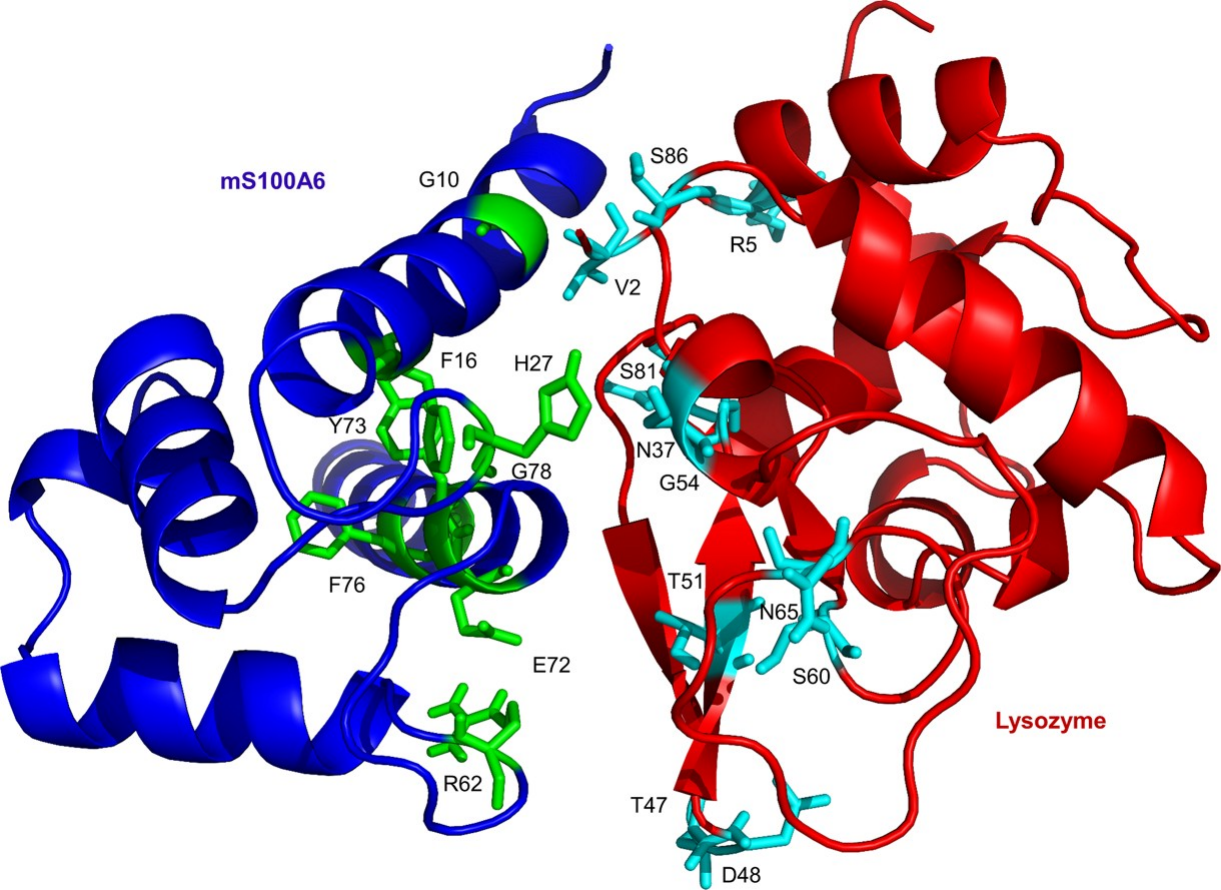
Representation of the 3D ribbon diagram of modeled lysozyme-mS100A6 complex. Lysozyme is painted in red and mS100A6 is painted in blue. The interacting residues of lysozyme and mS100A6 are shown in sticks representation in cyan and green, respectively.

Approximately 4000 structures of the complex were generated using procedure of rigid-body minimization in docking calculations. The top 200 structures with lowest energy scores were divided into 13 clusters and subjected to the analysis of water refinement [51,52]. The cluster one is the best amongst all which consist of 32 complex with lowest energy and RMSD value (Fig 3). The resulting 3D model of lysozyme-mS100A6 complex is shown in a ribbon representation in Fig 4. The interacting residues of lysozyme (V2, R5, N37, T47, D48, T51, G54, S60, N65, S81 and S86) are shown as sticks in cyan on the lysozyme structure, while those of mS100A6 (G10, F16, H27, R62, E72, Y73, F76 and G78) are shown as sticks in green on the mS100A6 structure.

### 3.4 Determination of binding site of labeled lysozyme with tranilast

**Fig 5:**
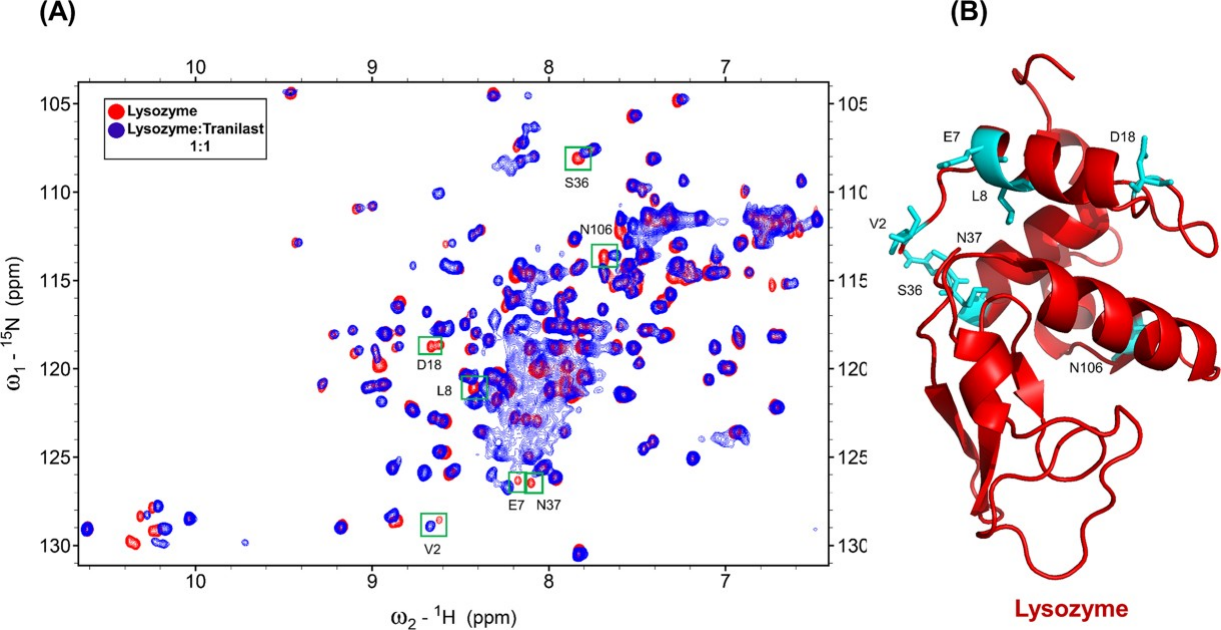
(A) Overlapped HSQC spectra of ^15^N-labeled lysozyme (red) and of ^15^N-labeled lysozyme with unlabeled tranilast (blue). Disappearing cross peak intensities are boxed in green. (B) The 3D ribbon diagram of lysozyme (red) shows the identified interacting residues to tranilast as sticks (cyan).

We also performed 2D NMR HSQC titration to find the binding site of lysozyme against tranilast. Fig 5A shows the superimposition of HSQC spectra of ^15^N labeled lysozyme and ^15^N labeled lysozyme complexed with tranilast. As it can be seen there are differences in HSQC signals (including complete disappearance of some cross peak intensities) after addition of tranilast. The HSQC signal of lysozyme complexed with tranilast in the interaction site was lower than of free lysozyme. This happens, most probably, due to the interaction between lysozyme and the drug (tranilast). The amino acids of lysozyme interacting with tranilast (V2, E7, L8, D18, S36, N37 and N106) are boxed in green.

We have found that the peaks corresponding to D18 and N106 also disappeared, however, these residues are located away from the binding site, this disappearance may happen due to flexibility and mobility of protein structure when the complex is formed. Residues D18 and N106 are present at the N- and C-terminal end of lysozyme sequence, respectively, and are surrounded by flexible loops.

### 3.5 Docking study of lysozyme with tranilast

**Fig 6:**
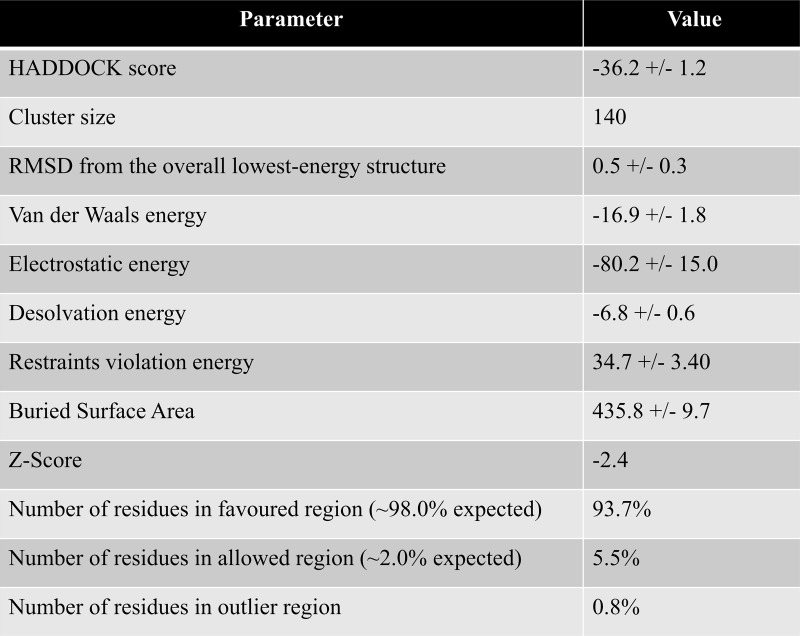
Haddock calculated parameter of the lysozyme-tranilast complex of cluster 1.

**Fig 7:**
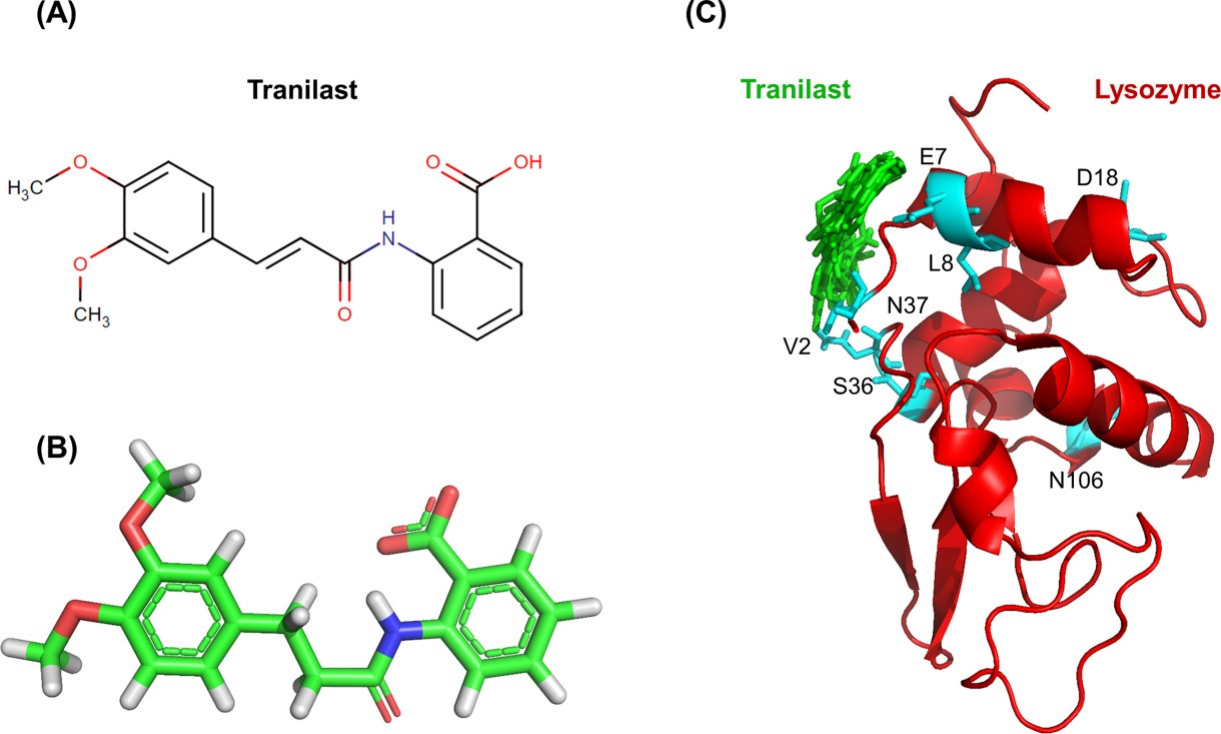
(A) Structural formula of tranilast. (B) 3D structure of tranilast. (C) Model of the lysozyme-tranilast complex; lysozyme (red) and a dozen possible structures of tranilast (green). The residues of lysozyme interacting with tranilast are shown as sticks in cyan and green, respectively.

To dock tranilast to lysozyme we used the method as described in “Docking study of lysozyme with mS100A6”. The residues with changed chemical shift in HSQC spectra were taken as constraints for the input parameters for the calculations of HADDOCK. The structure of tranilast was taken from the Drug Bank database (ID: DB07615). The final lysozyme-tranilast complex, taken from the lowest energy cluster 1. The 3D complex and docking result is shown in Figs 6 and 7C. The interacting residues of lysozyme (V2, E7, L8, D18, S36, N37 and N106) are shown as sticks in cyan on the lysozyme structure.

### 3.6 K_d_ of the lysozyme-mS100A6 and lysozyme-tranilast complexes

**Fig 8:**
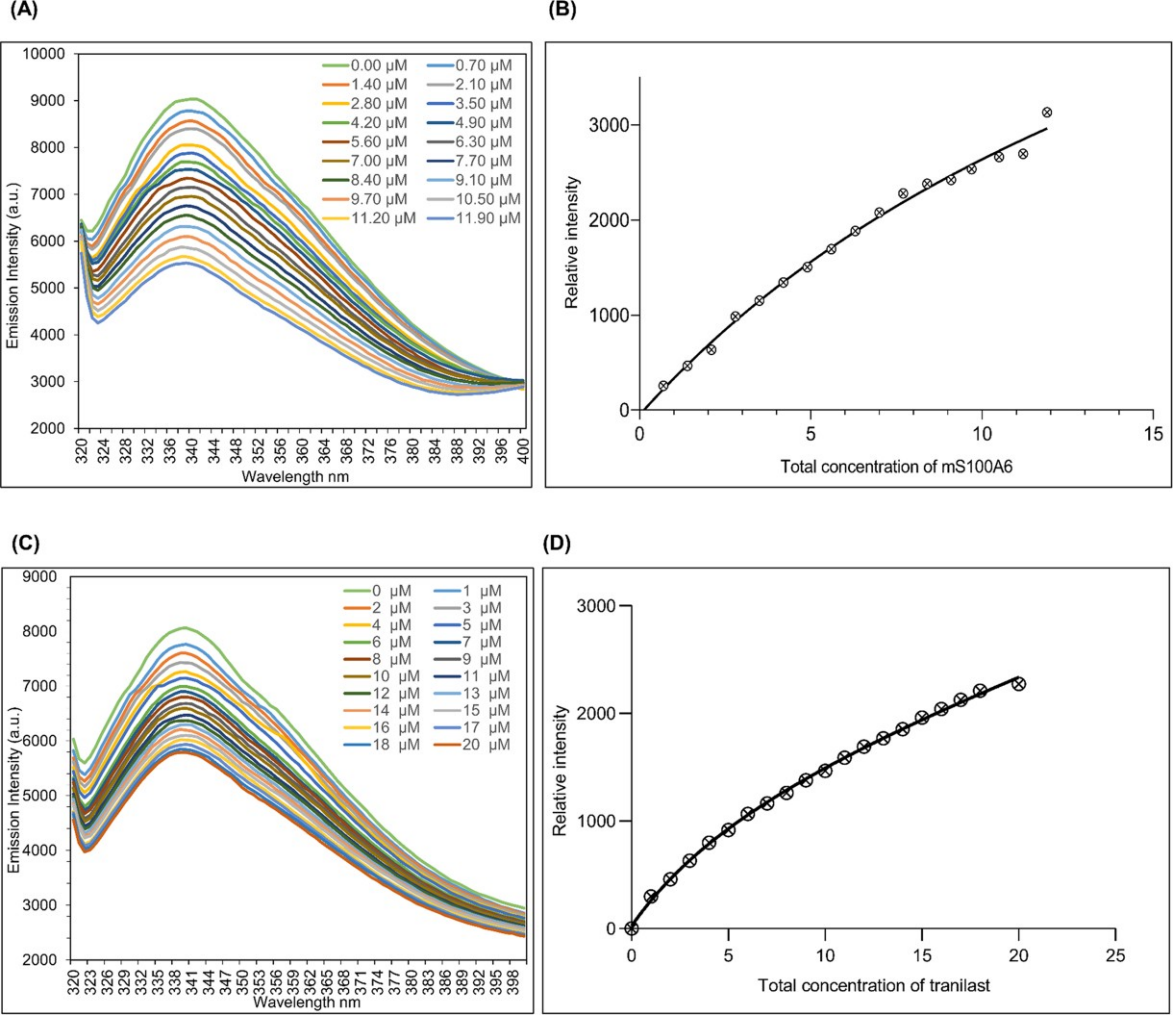
(A) Titration curve showing decreasing fluorescence intensities of lysozyme due to increasing concentration of mS100A6. (B) The nonlinear line of relative intensities against the total concentrations of mS100A6. (C) Titration curve of lysozyme decreasing fluorescence intensities against increasing tranilast concentration. (D) The nonlinear line of relative intensity against the total concentrations of tranilast.

Lysozyme contains six tryptophan residues in its sequence located at positions: Trp-28, Trp-62, Trp-63, Trp-108, Trp-111 and Trp-123. The analysis performed in NACCESS [53] shows that three tryptophan residues (Trp-62, Trp-63 and Trp-123) are exposed outside the lysozyme surface, while three others (Trp-28, Trp-108 and Trp-111) are buried inside the lysozyme structure. The excited wavelength of lysozyme is at 305 nm and the maximum emission is at 340 nm. To calculate K_d_, a decrease in fluorescence intensity (S1 and S2 Files) at 340 nm in the presence of mS100A6 was recorded (Fig 8A). Dissociation constant, K_d_, of the lysozyme-mS100A6 complex was calculated using the nonlinear curve fitting with the relative intensities against total concentration of the mS100A6, and was found to be 18±1.7 μM (Fig 8A and 8B). In a similar way, K_d_ of the lysozyme-tranilast complex was calculated and was found to be 6.7±0.9 μM (Fig 8C and 8D).

### 3.7 Proliferation of SW480 cancer cells

**Fig 9:**
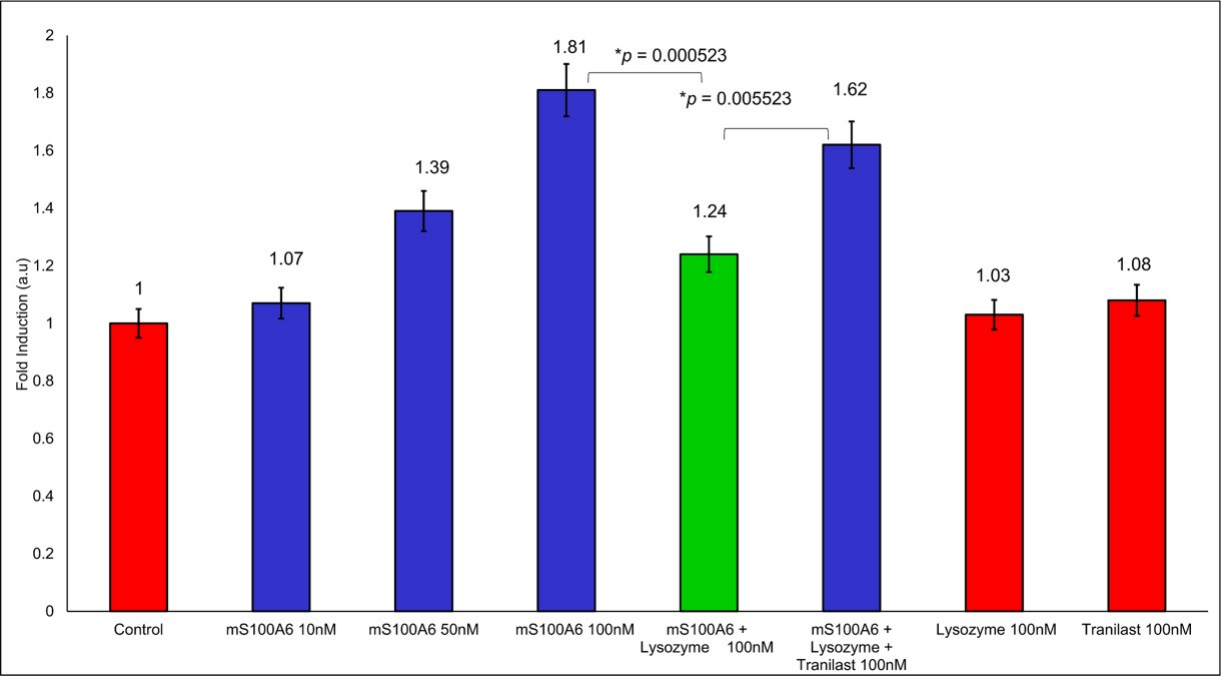
The WST1 assay. The bars represent the fold change in cell proliferation compared to control shown in red. The control represents proliferation of SW480 cells in the medium without mS100A6, lysozyme or tranilast and the *p* value of statistical analysis *(p ≤ 0*.*05)*.

While the concentration of mS100A6 rises to 10, 50, and 100 nM (Fig 9, lane 2, 3 and 4 in blue), the growth of SW480 cells, highly expressing RAGE, increases 1.81 fold (Fig 9, bar 4 from the left, blue). This may indicate that a signaling pathway leading to cell proliferation is activated in the presence of mS100A6. However, when the complex of lysozyme-mS100A6 was added to the medium of SW480 cells, a clear inhibition of the cell growth was observed since it dropped down to 1.24 (Fig 9, lane 5 in green). Such an observation indicates that lysozyme exhibits anti-proliferative properties. Furthermore, when tranilast was added to the SW480 cell medium containing lysozyme and mS100A6 cell proliferation increased 1.62 fold (Fig 9, lane 6 in blue). So, this is the first time reported data indicate that tranilast, when it binds to lysozyme and blocks its interaction with mS100A6, is able to restore cell proliferation (S3 File). We have also added the two controls of lysozyme and tranilast alone. There is no effect of 100 nM lysozyme alone as well as of 100 nM tranilast alone on cells. Tranilast has been shown to reduce cell proliferation and migration, and promotes apoptosis by different mechanisms, such as increase of AKT1 phosphorylation, decrease of ERK1/2 phosphorylation, and alterations in the cell cycle mediators to inhibit cell proliferation and migration; upregulation of p53 and caspase cascades to induce apoptosis [54,55]. Tranilast affected the S100A6/RAGE pathway-induced cell proliferation in our model. We cannot exclude other possible mechanisms of tranilast in our experiment. The concentrations of tranilast used to inhibit cell proliferation and migration, and to induce apoptosis in cancer cells were in the range of μM in previous studies. However, we only treated cancer cells with 100 nM of tranilast in our study, and there was no cytotoxicity under this condition, suggesting that the apoptotic signaling, such as p53 or caspase cascades, might not be induced in our condition.

## 4. Conclusion

**Fig 10:**
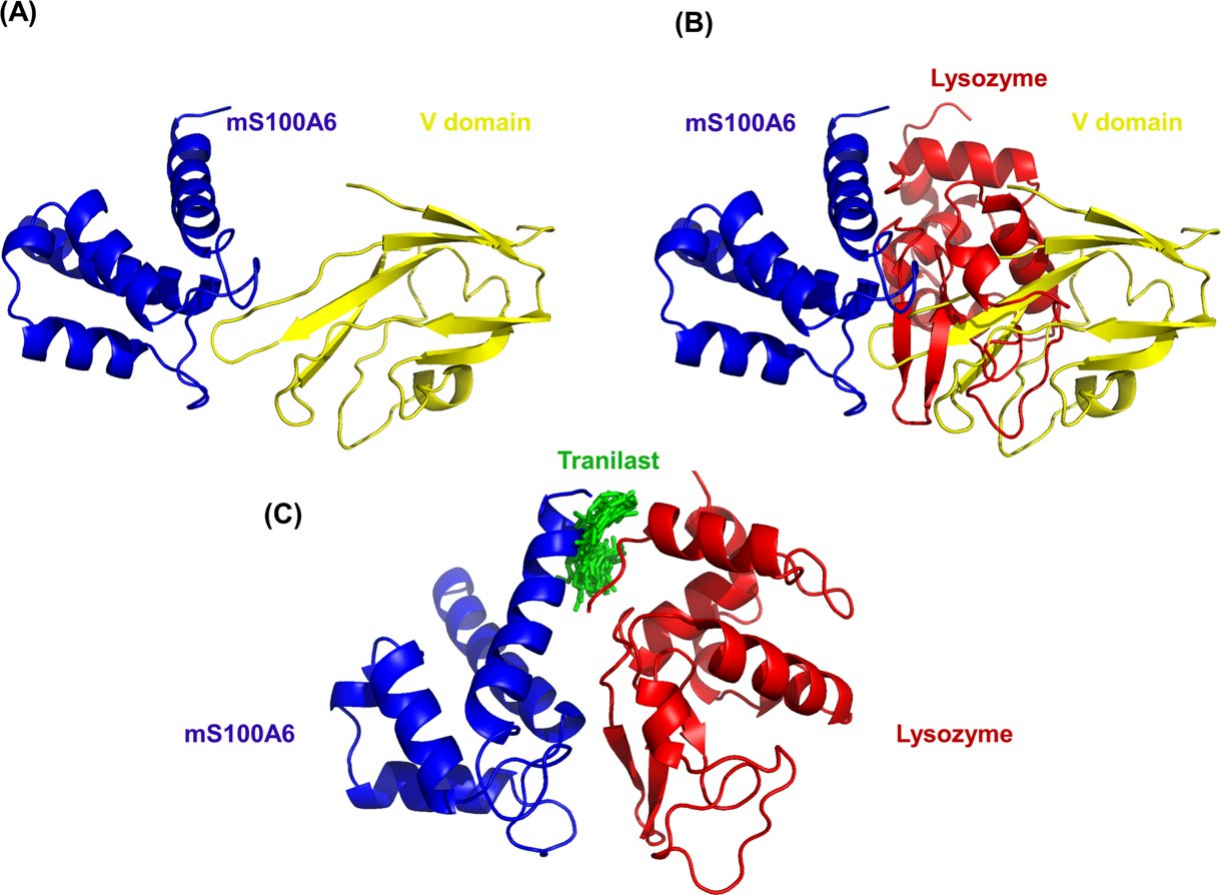
**(A) Structure of mS100A6-RAGE V domain complex** [26]**. (B) The lysozyme-mS100A6-RAGE V domain complex. (C) mS100A6-lysozyme complex with tranilast. S100A6 is in blue, lysozyme—in yellow and tranilast–in green**.

In this work, by studying mS100A6, lysozyme and tranilast, we have identified a novel way of controlling cell proliferation. We showed that lysozyme and its inhibitor, tranilast, might influence the inhibitory and stimulatory signaling pathways involved in this process. Firstly, we performed the NMR HSQC titration for labeled lysozyme with mS100A6, labeled lysozyme with tranilast, and labeled mS100A6 with lysozyme (Figs 1, 2 and 5). We have taken the amino acid constraints from HSQC spectra based on the chemical shift perturbation and used these constraints to perform docking studies. With the HADDOCK program, we generated structures of lysozyme-mS100A6 and lysozyme-tranilast complexes (Figs 4 and 7C, respectively). Using the fluorescence method, we calculated the dissociation constants, K_d_, of the lysozyme-mS100A6 and lysozyme-tranilast complexes (Fig 8). Finally, we performed the WST1 assay to prove that blocking the lysozyme-mS100A6 interaction with tranilast may lead to changes in activities of signaling pathways responsible for cell proliferation (Fig 9). We also demonstrate the putative structure of the lysozyme-mS100A6-tranilast complex using PyMOL program (Fig 10). The structure of mS100A6-RAGE V domain complex (Fig 10A) has been already reported by our group [26]. The lysozyme-mS100A6 structure overlapped with the structure of mS100A6-RAGE V domain indicates that lysozyme blocks interaction between mS100A6 and RAGE V domain (Fig 10B) and that tranilast blocks the interaction between mS100A6 and lysozyme (Fig 10C).

Thus, we conclude that lysozyme may inhibit cell proliferation whereas tranilast is able to restore cell growth. The studies presented in this work could be useful to understand the protein-protein and protein-drug interactions and the obtained results might be applied to design new therapies for treating disorders associated with cell proliferation such as cancers [29].

## Supporting information

S1 Fig mS100A6 protein marked on SDS-PAGE with molecular weight of 10.178 kDa. (TIF)

S2 Fig SDS-PAGE of lysozyme protein marked with molecular weight of 14.444.33 kDa. (TIF)

S3 Fig Analysis of the complex lysozyme-mS100A6 using the Ramachandran plot. (TIF)

S4 Fig Analysis of the complex lysozyme-Tranilast using the Ramachandran plot. (TIF)

S1 File Fluorescence intensity data analysis during the titration of lysozyme against mS100A6. (XLSX)

S2 File Fluorescence intensity data analysis during the titration of lysozyme against tranilast. (XLSX)

S3 File WST1 analysis of lysozyme, mS100A6 and tranilast. (XLSX)

## Abbreviations

NMR: Nuclear Magnetic Resonance
HSQC: Heteronuclear Single Quantum Coherence
HADDOCK: High Ambiguity Driven biomolecular DOCKing
IPTG: Isopropyl β-D-1-thiogalactopyranoside
AIRs: Ambiguous Interaction Restraints
PDB: Protein Data Bank
DTT: Dithiothreitol
K_d_: Dissociation Constant
HEWL: Hen Egg White Lysozyme
RAGE: Receptor for Advanced Glycation Endproducts
mS100A6: mutant S100A6(C3S)
WST-1: [2- (4-nitrophenyl) 4-[3-(4-iodophenyl)- 2H-5-tetrazolio]-1,3- benzene disulfonate)]
