# Supplementary figures and images for: Lysozyme as the anti-proliferative agent to block the interaction between S100A6 and the RAGE V domain
Source: PLoS One. 2019 May 9;14(5):e0216427. doi: 10.1371/journal.pone.0216427 (PMC6508705; doi:10.1371/journal.pone.0216427)

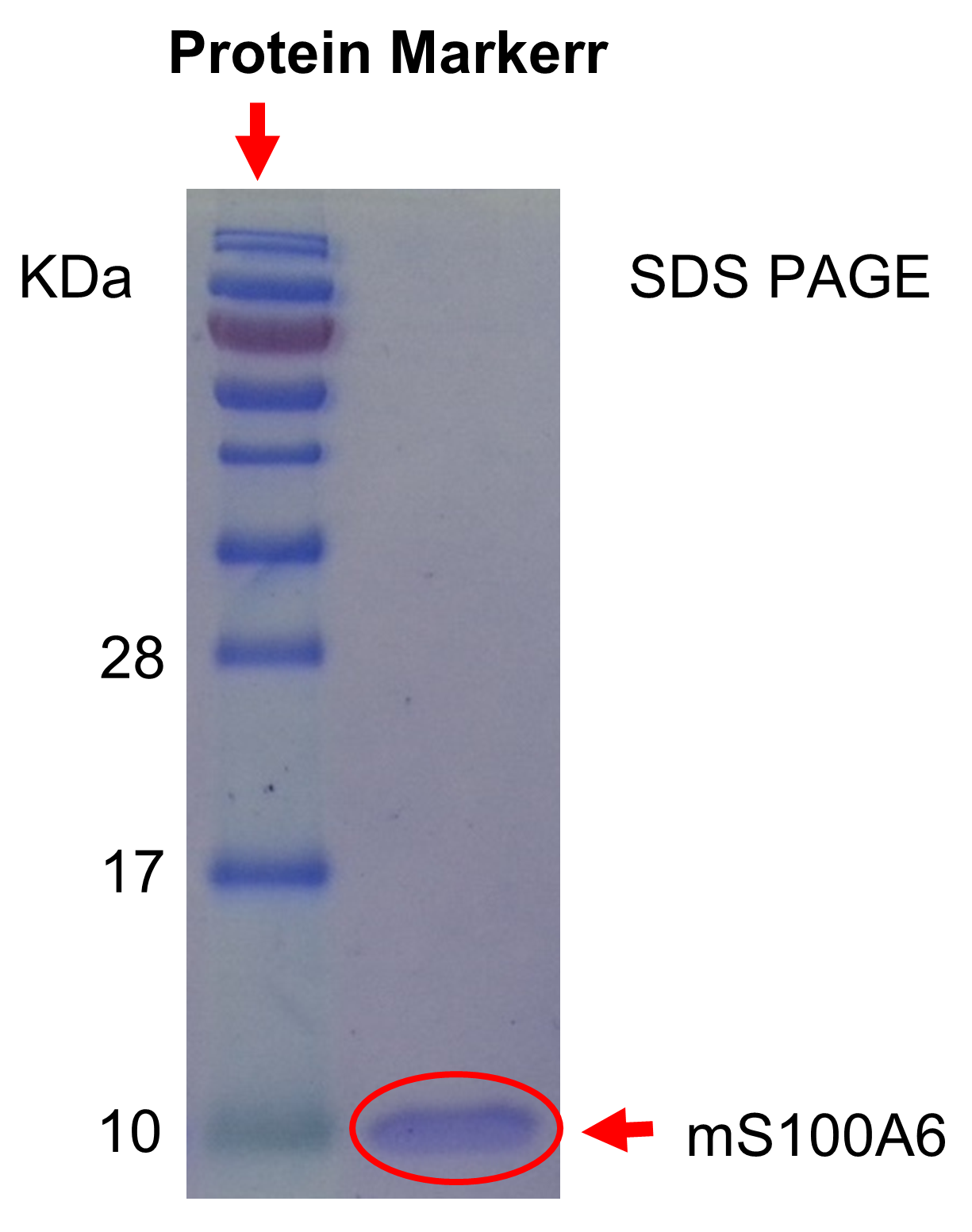

Supplement: S1 Fig — (TIF) [file pone.0216427.s001.TIF]

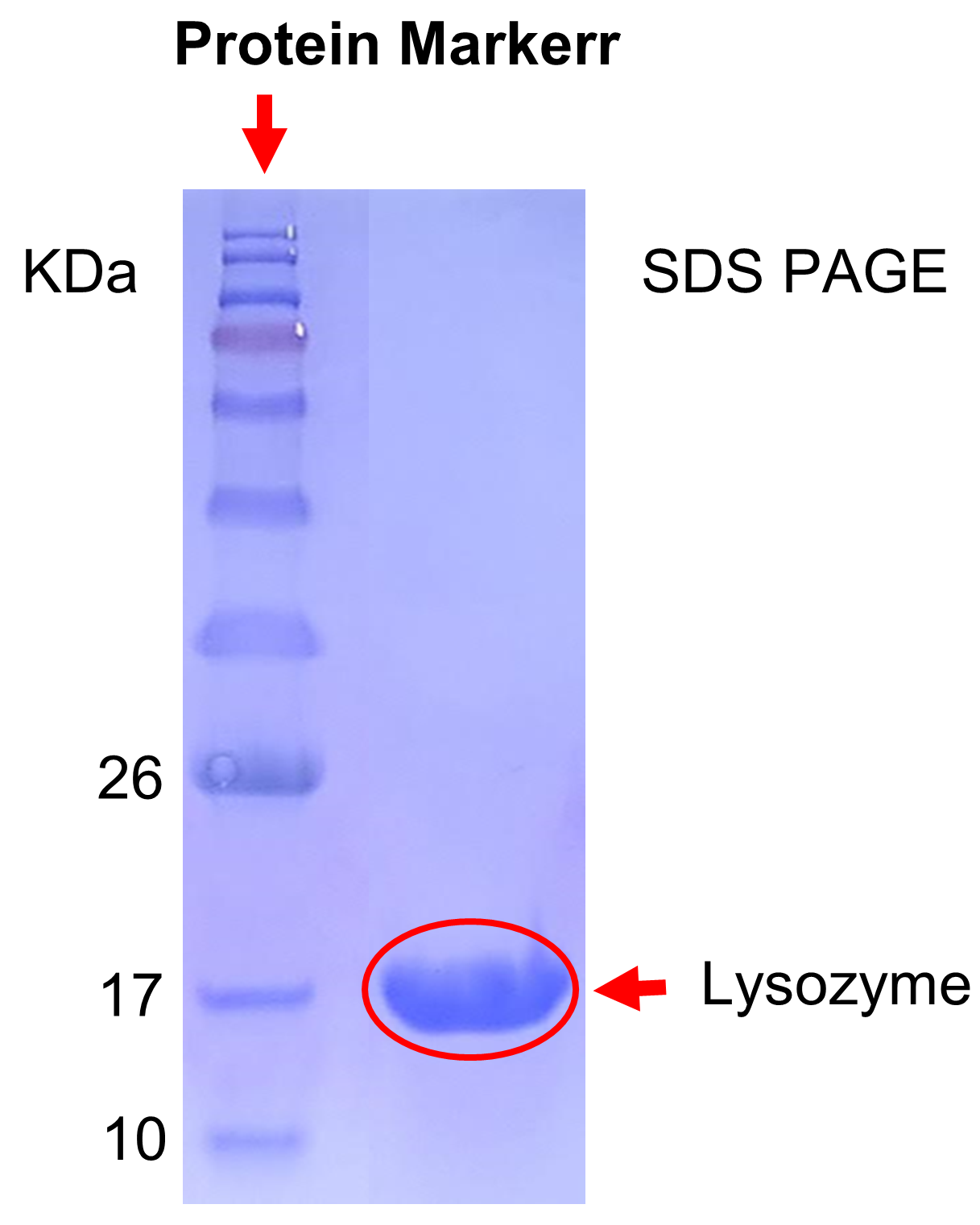

Supplement: S2 Fig — (TIF) [file pone.0216427.s002.TIF]

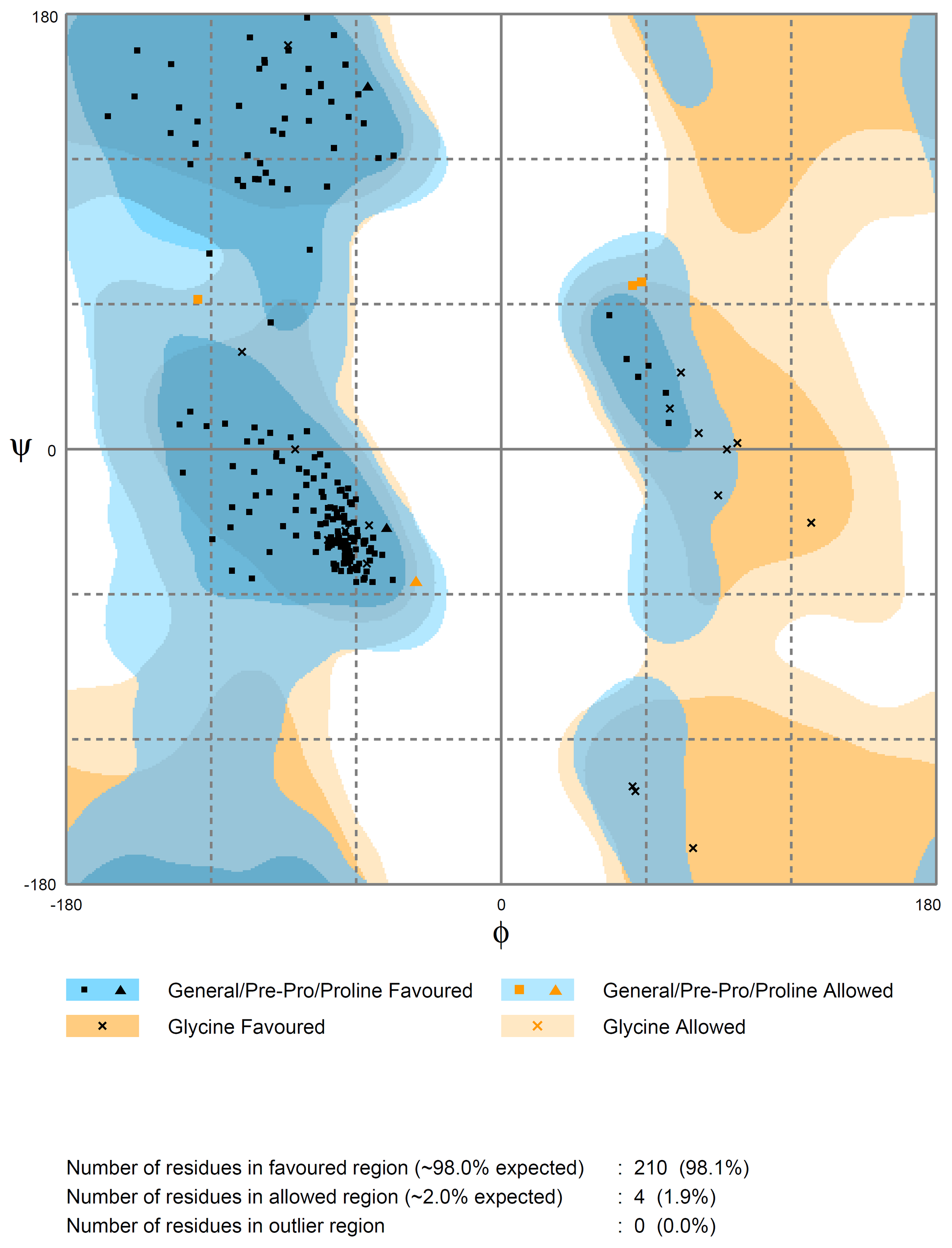

Supplement: S3 Fig — (TIF) [file pone.0216427.s003.TIF]

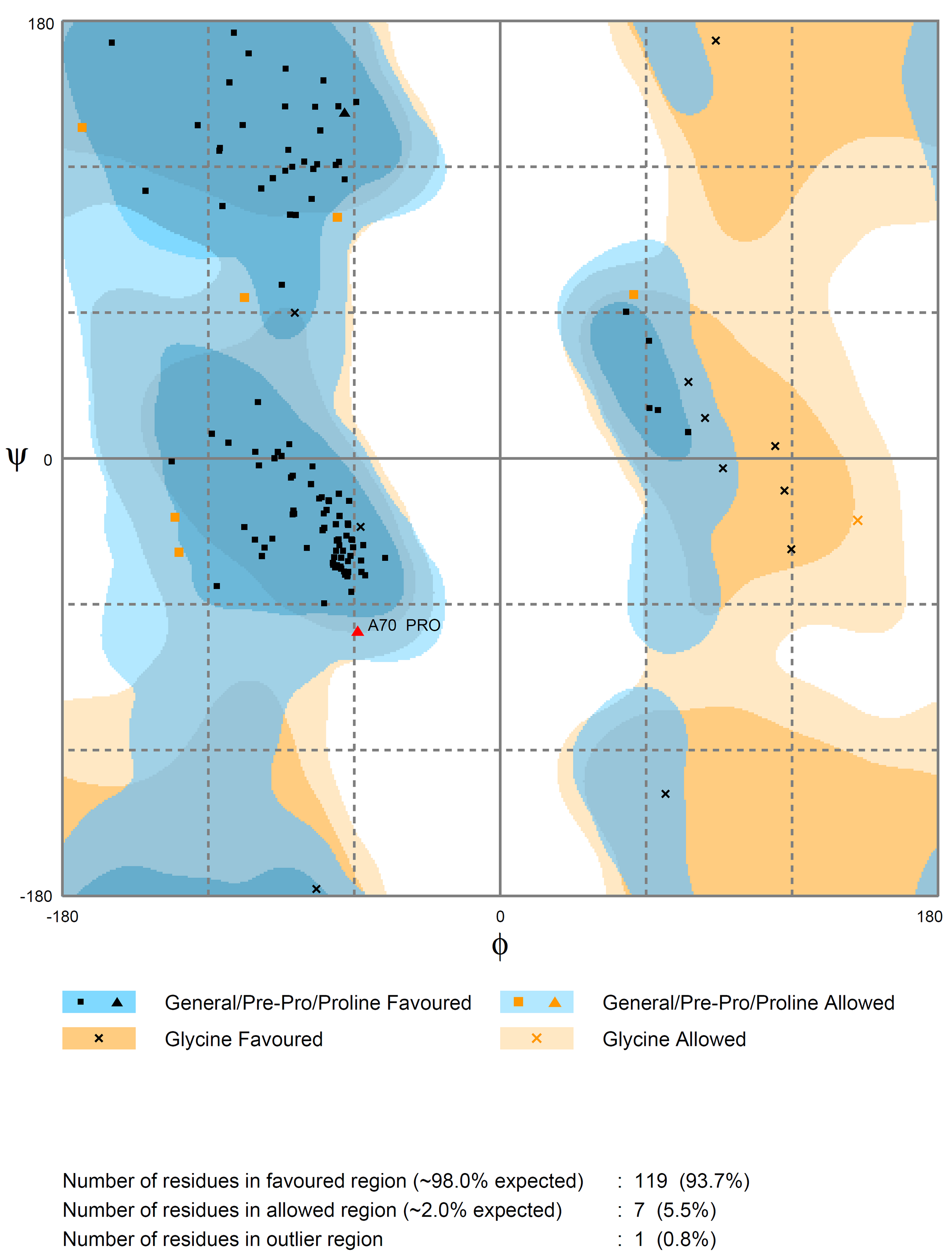

Supplement: S4 Fig — (TIF) [file pone.0216427.s004.TIF]
